# Translational Cross-Activation of the Encapsidated RNA of Potexviruses

**Published:** 2017

**Authors:** M. V. Arkhipenko, N. A. Nikitin, E. K. Donchenko, O. V. Karpova, J. G. Atabekov

**Affiliations:** Biology Department, Lomonosov Moscow State University, 1 bld. 12 Leninskie gory, Moscow, 119234 , Russia

**Keywords:** plant viruses, genomic RNA, cross-activation, potexviruses, translational activation, movement protein 1

## Abstract

We had shown the genomic RNA of potexviruses potato virus X and the alternanthera mosaic virus to be inaccessible *in vitro *to ribosomes while in intact virion form, but the RNAs can be translationally activated following the binding of movement protein 1 (MP1) to virus particles. Here, we present the results of the follow-up study targeting two more potexvirus species – the Narcissus mosaic virus and the Potato aucuba mosaic virus. We found encapsidated potexviral RNA to share common translational features *in vitro *and the MP1 to be potent over homological virions of its “own” species and over heterological virions of other species, as well exhibiting selective specificity. Reciprocal cross-activation is observed among viral species phylogenetically either close or distant. There is direct evidence that MP1 binding to the end of the virion is necessary, but not sufficient, for translational activation of encapsidated RNA.

## INTRODUCTION

We have shown in our previous study that the genomic RNA of the Potexvirus-belonging potato virus X (PVX) is inaccessible to ribosomes in intact virions *in vitro* while being rendered into translatable form upon phosphorylation of the PVX coat protein (CP), forming the virion or binding to PVX movement protein 1 (MP1). We suggest that two mechanisms of translation activation operate at different stages of the infection. The encapsidated RNA becomes accessible to ribosomal translation in primary infected cells following the phosphorylation of the PVX coat protein. The MP1 produced during the course of the infection binds to the PVX virions’ end, producing the virus transport form and, thus, activating the encapsidated RNA [1-3].

We clarified subsequently that MP1 interacts with the terminal CP molecules of the PVX helical virions corresponding to the 5′ end of PVX RNA, while not contacting the genomic RNA itself. Phosphorylation and the MP1 interaction engage different regions of the PVX CP: the former targets the 19 N-terminal amino acids, whereas the latter is restricted to the 10/18 amino acids long C-terminal fragment accessible to the MP1 at the end of the virion [4, 5].

The MP1 binding to intact PVX virions results in a destabilization of the entire helical PVX structure, leading in turn to a conformational shift from the stable non-translatable form to a metastable form, where the 5’ end of the PVX RNA is accessible to ribosomes. So, the MP1, being a component of the PVX transport form, may be perceived as a mediator of the virion-packed genomic RNA translation [1, 6, 7].

A set of deletion mutants was used to reveal the virion-binding MP1 motif located between the amino acid positions 112 and 122. Noteworthy, the MP1-CP interaction is necessary but not sufficient to translationally activate encapsidated RNA. The MP1-dependent translational activation is abolished upon the removal of a MP1 fragment not interfering with virion binding or the protein phosphorylation, probably due to conformational changes in the protein molecule [5].

The genomic RNA of another potexvirus, the alternanthera mosaic virus (AltMV, strain AltMV*-*MU), is non-translatable *in vitro* in the intact virion but, similar to PVX RNA, can be rendered translatable by the phosphorylation of the virion coat protein or by interaction with AltMV MP1 [8, 9]. The observation of such a similarity raised a question: whether PVX MP1 can activate genomic virion-packed AltMV RNA and vice versa. It was shown that encapsidated AltMV RNA is efficiently translationally activated by PVX MP1 [8]. Moreover, the opposite proved true also – the virion-packed PVX RNA is translationally activated by AltMV MP1 [10].

One may consider, based on the presented data, the translational features of encapsidated RNA, as well as the translation activation pathway, to be shared across the genus *Potexvirus*.

The present study deals with the translational features of two more potexviruses: the narcissus mosaic virus (NMV) and the potato aucuba mosaic virus (PAMV). We looked into whether the corresponding MP1s are capable of translationally activating the encapsidated RNA of the four *Potexvirus *members: namely, PVX, AltMV, NMV, and PAMV.

## MATERIALS AND METHODS

**Virus isolation and viral RNA extraction**

The PVX, NMV, and PAMV preparations were isolated from infected *Datura stramonium* L. plants as described in [1]. The AltMV preparations were isolated from infected *Portulaca grandiflora *plants as previously described in [8]. The viral RNA was prepared using the phenol method with modifications [11].

**Production of mutant MP1**

Recombinant PVX and AltMV MP1 molecules were constructed as described in [1, 10].

The NMV MP1 and PAMV MP1 expressing plasmids were constructed using the pQE30 vector (Qiagen). The corresponding coding regions, supplemented with His6 tags, were amplified on the the NMV and PAMV viral RNA templates using the following primers: NMV-forward-BamHI(+) 5’-acacggatccatggactgtaagta-3’, NMV-reverse-PstI(-) 5’-acacctgcagcgtagttaacaggtg-3’ Auc-forward-BamHI(+) 5’-acatggatccggaatggaatat-3’, and Auc-reverse-PstI(-) 5’-acacctgcagatcagtctaaat-3’. The *Escherichia coli *M15 [pREP4] strain was transformed with the constructs. The recombinant proteins were purified following expression induction by chromatography on a Ni-NTA agarose. The SDS PAGE analysis of the protein samples using a 8-20% gel revealed a single band corresponding to either NMV MP1 (26.7 kDa) or PAMV MP1 (27.2 kDa).

**Translation in vitro**

The translation was performed in a wheat germ extract cell-free system (Promega), following a modified manufacturer protocol as described [12]. The RNA input was 40 µg/µl. The study of translational activation was performed with a PVX to recombinant MP1 molar ratio of 1 : 100, i.e., 1 µg RNA (20 µg of virus) per 1.4 µg MP1.

**Immune electron microscopy**

The immune electron microscopic observation was done as described in [13]. Polyclonal antisera to the PVX MP1, AltMV MP1, and PAMV MP1 were used as primary antibodies according to [10]. Gold-conjugated (12 nm) antibodies were used as secondary antibodies. The samples were contrasted with 2% aqueous uranyl acetate. The stained samples were examined under a JEOL JEM-1011 transmission electron microscope (JEOL, Japan) at 80 kV. Images were acquired using a Gatan Erlangshen ES500W digital camera and the Gatan Digital Micrograph software.

## RESULTS AND DISCUSSION

**Fig. 1:**
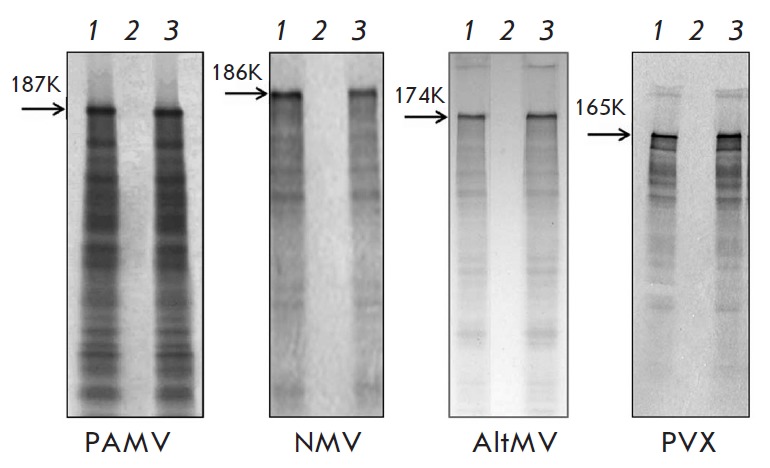
Translational activation of encapsidated PAMV and NMV RNA *in vitro*. PAMV (A), NMV (B), AltMV, (C) and PVX (D) genomic RNA (lanes 1); encapsidated RNA (lanes 2); encapsidated RNA incubated with MP1 (lanes 3). The arrowheads indicate the position of the PAMV replicase (187K), NMV replicase (186K), AltMV replicase (174K), and PVX replicase (165K). Electrophoretic analysis of ^35^S-labeled translation products.

To study the translational features of *Potexvirus *encapsidated RNA, we propagated, harvested, and purified NMV and PAMV viral preparations, accompanied by these viruses’ recombinant MP1 samples. We found the translational properties of encapsidated NMV and PAMV RNA to not differ from those of PVX and AltMV. Our findings show virion-packed NMV and PAMV RNA to be nontranslatable *in vitro* (*Fig. 1, lane 2*), but it could be rendered translatable following exposure to its own MP1 (*Fig. 1, lane 3*). The same had been demonstrated for AltMV and PVX RNA (*Fig. 1, positive controls*).

**Fig. 2:**
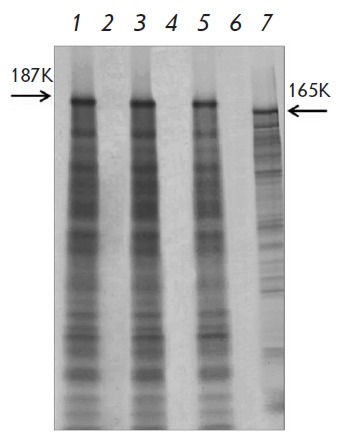
Translational activation of genomic PAMV RNA within viral particles *in vitro*. PAMV genomic RNA (lane 1); encapsidated PAMV RNA (lane 2); encapsidated PAMV RNA incubated with MP1 of PAMV (lane 3), PVX MP1 (lane 4), NMV MP1 (lane 5); and PVX-viral-particles-incubated PAMV MP1 (lane 6) or PVX MP1 (lane 7). The arrowheads indicate the position of the PAMV replicase (187K) and PVX replicase (165K). Electrophoretic analysis of ^35^S-labeled translation products.

We reported previously on the translational cross-activation of encapsidated AltMV and PVX RNA after the interaction of PVX with AltMV MP1 and vice versa [8, 10]. We aimed to further investigate in this study whether cross-activation exists among other potexviruses. Our experiments revealed that PAMV virion-packed RNA can be rendered translationally active upon exposure to NMV MP1 (*Fig. 2, 5*). Worthwhile, the treatment of encapsidated PAMV RNA with PVX MP1 (*Fig. 2, 4*) does not render it translationally active contrary to AltMV RNA [8]. The same is true in the inverse situation: PVX virion-packed RNA treated with PAMV MP1 remains inaccessible to ribosomes (*Fig. 2, 6*).

**Fig. 3:**
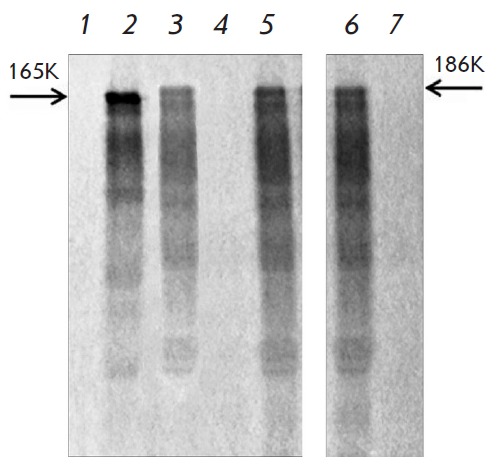
Translational activation of genomic NMV RNA within viral particles *in vitro*. Encapsidated NMV RNA (lane 1); encapsidated NMV RNA with PVX MP1 (lane 3), NMV MP1 (lane 5), and AltMV MP1 (lane 7). Purified NMV genomic RNA genome as a positive control (lane 6). Translation of encapsidated PVX RNA incubated with NMV MP1 (lane 4) and PVX MP1 as a positive control (lane 2). The arrowheads indicate the position of NMV (186K) and PVX (165K) RNA polymerases. Electrophoretic analysis of ^35^S labeled translation products.

**Fig. 4:**
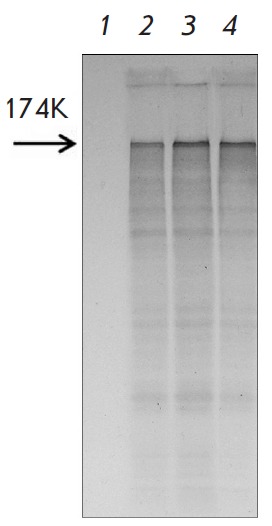
Translational activation of encapsidated AltMV RNA *in vitro*. AltMV virion-packed RNA (lane 1); AltMV genomic RNA as a positive control (lane 2); encapsidated AltMV RNA with PVX MP1 (lane 3) or NMV MP1 (lane 4). The arrowhead indicates the position of the AlMV replicase (174K). Electrophoretic analysis of ^35^S-labeled translation products.

A different pattern was observed when analyzing the translation activation of NMV and PVX virion-packed RNA, while NMV MP1 appeared incapable of activating PVX RNA (*Fig. 3, 4*), whereas PVX MP1 activated encapsidated NMV RNA (*Fig. 3, 3*). No reciprocal cross-activation exists between NMV and PVX, as is the case for NMV and AltMV; i.e., NMV virion-packed RNA remains nontranslatable when exposed to AltMV MP1 (*Fig. 3, 7*); AltMV virion-packed RNA, in contrast, does undergo activation under treatment with NMV MP1 (*Fig. 4, 4*) or PVX MP1 (*Fig. 4, 3*) [8]. These observations are the first pieces of evidence of nonreciprocal cross-activation of encapsidated potexviral RNA.

We also tested whether PAMV MP1 is capable of activating encapsidated NMV or AltMV RNA. This movement protein proved to activate the translation of NMV viral RNA but not AltMV viral RNA. Similarly, the PAMV virion-packed RNA does not translationally become activated upon treatment with AltMV MP1 (data not shown).

The results described above are summarized in Table 1.

**Table:** Potexvirus-encapsidated RNA translationally activated by MP1.

Virus	Protein			
	PVX MP1	NMV MP1	PAMV MP1	AltMV MP1
PVX	+	-	-	+
NMV	+	+	+	-
PAMV	-	+	+	-
AltMV	+	+	-	+

Note. The color indicates the phylogenetic subgroups of MP1 according to Wong et al. [16]: Ia (pink); Ib (green); Ic (yellow). “+” – translational activation, “-” – no translational activation.

**Fig. 5:**
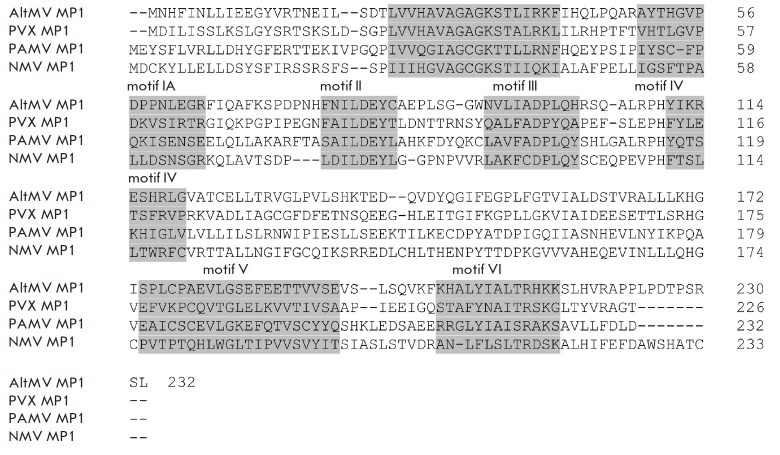
Alignment of the amino acid sequences of AltMV MP1, PVX MP1, PAMV MP1, and NMV MP1. The grey shading denote the MP1 conservative motifs (motifs I, IA, II, III, IV, V, VI) [15].

The findings of cross-activation studies point out that NMV MP1 is capable of translationally activating encapsidated PAMV and AltMV RNA but not encapsidated PVX RNA. Furthermore, PAMV MP1 can translationally activate NMV virion-packed RNA but not AltMV and PVX RNA. We have already observed cross-activation of encapsidated AltMV and PVX RNA. Now, we have found evidence of PVX MP1 activating NMV virion-packed RNA, as well as encapsidated AltMV RNA, but not PAMV RNA. Moreover, AltMV MP1 was found to lack the capability of activing PAMV and NMV virion-packed RNA (*Table*). Reciprocal cross-activation was discovered in pairs: PVX – AltMV and NMV – PAMV.

Broadly, potexviral movement proteins were shown to be able to translationally activate the encapsidated RNA of kin species showing, however, selective specificity.

The amino acid sequence of the corresponding MP1 was compared to shed some light on the data obtained (*Fig. 5*). *Potexvirus* movement proteins are known to belong to superfamily I helicases, which contain seven highly conservative NTPase/RNA helicase motifs constituting a NTPase/RNA helicase domain [14, 15]. We have previously produced a set of PVX MP1 variants that carry deletions in different functional regions. The deletions proved to be negligible in regard to the MP1-CP interaction unless those involve the motif IV of the NTPase/RNA helicase domain (amino acid residues 112–122). Hence, our sequence analysis considered mainly motif IV [5].

The MP1 motif IV sequences of PVX, AltMV, PAMV, and NMV show a high degree of variability: hence, the prediction that these MP1s bind to the end of heterologous virions in the absence of cross-activation is questionable (*Fig. 5*). Additional experiments were performed to clarify this question.

**Fig. 6:**
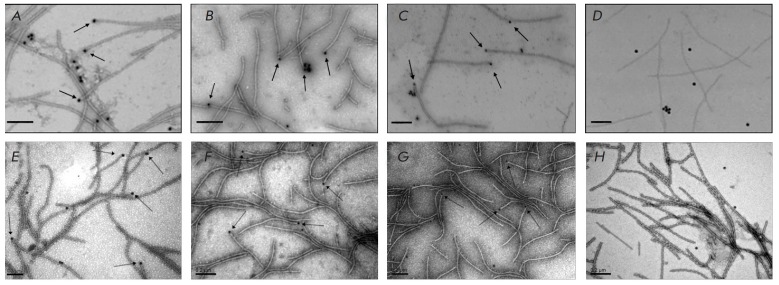
Immunoelectron microscopy of PVX virions interaction with PVX MP1 (A), AltMV MP1 (B), or PAMV MP1 (C) and AltMV virions interactions with AltMV MP1 (E), NMV MP1 (F), or PAMV MP1 (G). Primary antibodies against PVX MP1 (A – positive control), AltMV MP1 (B,E), PAMV MP1 (C,G), or NMV MP1 (F) and secondary gold-conjugated (12 nm) antibodies. Negative controls: PVX (D) or AltMV (H) virions treated with primary and secondary gold-labeled antibodies in the absence of MP1. The arrowheads indicate the position of MP1 bound to the end of virions. The scale bars represent 200 nm.

We used immune electron microscopy employing primary antibodies against the MP1 of PVX, AltMV, and PAMV and gold-conjugated secondary antibodies. This approach helped us clearly visualize the binding to the PVX virion end of PVX MP1 (*Fig. 6A*, positive control) and AltMV MP1 (*Fig. 6B*) that render encapsidated PVX RNA translatable, as well as the PAMV MP1 that lacks such capability (*Fig. 6C*). In a control experiment (*Fig. 6D, negative control*), no binding of gold beads was observed in the absence of MP1. Similar results were obtained when studying AltMV viral particles (*Fig. 6E–M*): virion end binding was observed for PAMV MP1 (*Fig. 6G*), AltMV MP1 (*Fig. 6E*), and NMV MP1 (*Fig. 6F*), while only AltMV MP1was able to activate AltMV virion-packed RNA.

Our results provide the first piece of direct evidence of MP1 physical binding to a heterologous virion end. Such binding is necessary, but not sufficient, to the translational activation of encapsidated RNA.

A phylogenetic analysis of potexviral MP1 [16] split the *Potexvirus *genus members into three subclusters, Ia, Ib, and Ic. AltMV MP1 was assigned to Ia; NMV and PAMV MP1, to Ib; and PVX MP1, to Ic.

We have demonstrated in this study a reciprocal cross-activation of translation in the pairs PAMV-NMV and AltMV-PVX, while other pairs show either non-reciprocal cross-activation (NMV-PVX and NMV-AltMV) or a total absence of cross-activation (PAMV-PVX) (*Table*). The PAMV and NMV clustering in the same subgroup, Ib, tracks well with the fully reciprocal cross-activation observed for this pair. The observed cross-activation of PVX and AltMV virion-packed RNA corresponds probably to a lesser branching between the subgroups Ia and Ic compared to the one between Ia and Ib [16]. We suggest that MP1 conformation and interaction with CP C-terminal region are key features that determine the specificity of translational activation. We suggest that the MP1-CP interaction destabilizes the proteinaceous helical virion. The activation that is abolished by MP1 phosphorylation further supports this assumption [5]. Our previous data on MP1 deletion mutants successfully binding to a virion does not support the idea of the MP1 conformation being crucial to virion binding [5]. In addition, Rodionova *et al*. showed that the full-length movement proteins of potexviruses can bind to heterologous virions but fail to activate the translation of its genomic RNA. We consider phylogenetically related movement proteins to share conformational features and, thus, able to loosen the helical proteinaceous virion of either virus, hence exposing the 5’-end of genomic RNA to ribosomes [7].

## CONCLUSIONS

Our results clearly indicate that encapsidated potexviral RNAs share translational features *in vitro*. Direct evidence of MP1 binding to the virion end being essential but not sufficient to induce translation of genomic RNA was obtained. Potexviral movement proteins 1 are capable of translationally activating heterologous potexviruses virion-packed RNA with unequal specificity. Reciprocal cross-activation is observed for potexviruses of the same subgroup (NMV–PAMV, subgroup *Ib*) or closely related subgroups (PVX – AltMV, subgroups *Ic* and *Ia*). The movement proteins 1 capable of translationally activating heterologous potexviruses encapsidated RNA are likely to share protein molecule conformation; this suggestion, however, is a matter for further research.

## Abbreviations

CP: coat protein
PAMV: potato aucuba mosaic virus
AltMV: alternanthera mosaic virus
NMV: narcissus mosaic virus
MP1: movement protein 1
PVX: potato virus X
